# Novel mutations in the VKORC1 gene of wild rats and mice – a response to 50 years of selection pressure by warfarin?

**DOI:** 10.1186/1471-2156-10-4

**Published:** 2009-02-06

**Authors:** Simone Rost, Hans-Joachim Pelz, Sandra Menzel, Alan D MacNicoll, Vanina León, Ki-Joon Song, Thomas Jäkel, Johannes Oldenburg, Clemens R Müller

**Affiliations:** 1 Department of Human Genetics, University of Wuerzburg, Wuerzburg, Germany; 2 Federal Research Centre for Cultivated Plants – Julius Kuehn-Institute, Vertebrate Research, Toppheideweg 88, 48161 Muenster, Germany; 3 Central Science Laboratory, Sand Hutton, York, UK; 4 Department of Ecology, Genetics and Evolution, Faculty of Exact and Natural Sciences, Buenos Aires University, Buenos Aires, Argentina; 5 Department of Microbiology, College of Medicine, Bank for Pathogenic viruses, Korea University, Seoul, Korea; 6 GTZ Office Bangkok, 193/63 Lake Ratchada Bldg., 16th Floor, New Ratchadapisek Road, Bangkok 10110, Thailand; 7 Institute of Experimental Haematology and Transfusion Medicine, University of Bonn, 53105 Bonn, Germany

## Abstract

**Background:**

Coumarin derivatives have been in world-wide use for rodent pest control for more than 50 years. Due to their retarded action as inhibitors of blood coagulation by repression of the vitamin K reductase (VKOR) activity, they are the rodenticides of choice against several species. Resistance to these compounds has been reported for rodent populations from many countries around the world and poses a considerable problem for efficacy of pest control.

**Results:**

In the present study, we have sequenced the *VKORC1 *genes of more than 250 rats and mice trapped in anticoagulant-exposed areas from four continents, and identified 18 novel and five published missense mutations, as well as eight neutral sequence variants, in a total of 178 animals. Mutagenesis in *VKORC1 *cDNA constructs and their recombinant expression revealed that these mutations reduced VKOR activities as compared to the wild-type protein. However, the *in vitro *enzyme assay used was not suited to convincingly demonstrate the warfarin resistance of all mutant proteins

**Conclusion:**

Our results corroborate the *VKORC1 *gene as the main target for spontaneous mutations conferring warfarin resistance. The mechanism(s) of how mutations in the *VKORC1 *gene mediate insensitivity to coumarins *in vivo *has still to be elucidated.

## Background

Coumarin derivatives, e.g. warfarin, act as vitamin K-antagonists and are in world-wide use as anticoagulants for therapy and prophylaxis of thrombotic diseases in humans, and as rodenticides for pest control. The physiological target of coumarins is the endoplasmic enzyme vitamin K-epoxide reductase (VKOR) [1,2]. A key component of the VKOR was recently identified and named VKORC1 [3,4]. Mutations in VKORC1 have been shown to cause two different hereditary phenotypes: warfarin-resistance (OMIM #122700) and defective blood coagulation owing to vitamin K-dependent coagulation factor deficiency type 2 (VKCFD2; OMIM #607473) [3]. The vitamin K-cycle provides vitamin K-hydroquinone, the essential cofactor for the γ-glutamyl carboxylase catalysing the post-translational modification of the vitamin K-dependent proteins [5]. These proteins are involved in blood coagulation (factor II, VII, IX, X, Protein S, C and Z), cell cycle regulation (growth-arrest specific protein 6) and bone metabolism (osteocalcin and matrix gla-protein) [6,7]. Coumarins inhibit blood coagulation by suppressing VKOR activity and consequently γ-carboxylation of vitamin K-dependent proteins.

Warfarin is a first-generation anticoagulant and has been used for pest control since the 1950's. Resistance to this coumarin derivative was first observed in *Rattus norvegicus *in the UK in 1958 [8] and led to the development of a more potent "second-generation" of anticoagulants such as difenacoum, bromadiolone and brodifacoum in the 1970's and 1980's. But soon after their introduction, resistance to specific second generation compounds was also observed in rodents [9-13].

In a previous publication, we have reported on a series of mutations in the *VKORC1 *gene in warfarin resistant rat and mouse populations from different areas in Europe [14]. This report is an update of the mutation study presenting novel mutations and common polymorphisms found in rodents from anticoagulant-exposed areas in Europe, South Africa, East Asia and both North and South America. The different mutations were recombinantly expressed in a human cell line and their VKOR activities and inhibition by warfarin were studied.

## Results and Discussion

More than 250 rats and mice from anticoagulant-exposed areas in Europe, East Asia, South Africa and both North and South America were screened for mutations in the VKORC1 gene. Pre-screening by ARMS-PCR for the Tyr139Cys mutation and subsequent sequence analysis of all three exons and flanking intronic regions revealed a panel of mutations and SNPs (single nucleotide polymorphisms) in the *VKORC1 *gene (Tables 1 and 2). Most of these mutations have not been described before.

**Table 1:** VKORC1 mutations and polymorphisms found in *Rattus norvegicus *from different geographic areas.

**Geographic area**		**Amino acid substitutions**	**No. of samples**	**Silent mutations**
**England**	Cambridge/Essex	Phe63Cys, Ala26Thr	1	Ile82Ile
		Phe63Cys, Tyr39Asn	1	Ile82Ile
		Phe63Cys	15	Ile82Ile
	Norfolk	Tyr139Cys	1	Ile82Ile
		Tyr139Cys	1	--
	Gloucestershire	Tyr139Cys	1	--
	Lincolnshire	Tyr139Cys	2	Ile82Ile
	Shropshire	Tyr139Ser	2	--
	Lancashire	Leu128Gln	2	--
	Nottinghamshire	Arg33Pro	2	--
				
**Hungary**	Békés	Tyr139Cys	1	Ile82Ile
		--	2	Ile82Ile
	Maglód	Tyr139Cys	9	not investigated
				
**Azores**	Terceira	Ile90Leu, Val112Leu	2	Leu94Leu, Ile107Ile, Thr137Thr, Ala143Ala
		Ile90Leu	1	Leu94Leu, Ile107Ile, Thr137Thr, Ala143Ala
		Ile90Leu	1	Ile107Ile, Thr137Thr
		--	12	Ile82Ile
				
**Korea**	Seoul	Tyr139Phe	6	--
		Tyr139Phe, Ala21Thr	1	--
		--	1	Ile82Ile
				
**Indonesia**		--	3	Ile82Ile
		Ile90Leu	5	Ile107Ile, Thr137Thr
		Ile90Leu, Ala143Val	1	Ser103Ser, Ile107Ile, Thr137Thr
		Ile90Leu, Ile141Val, Ala143Val	1	Ser103Ser, Ile107Ile, Thr137Thr
		Ile90Leu, Ile141Val	6	Ile107Ile, Thr137Thr
				
**Thailand**		Ala143Val	2	Ser103Ser, Ile107Ile, Thr137Thr
				
**Japan**		Glu67Lys	6	--
				
**USA**	Santa Cruz	Ile90Leu	3	Arg12Arg, Leu94Leu, Ile107Ile, Thr137Thr, Ala143Ala
	Chicago	Arg35Pro	6	--
		Arg35Pro	8	Ile82Ile
		--	4	Ile82Ile
				
**Argentina**	Buenos Aires	Trp59Arg, Ile90Leu	7	Arg12Arg, Leu94Leu, Ile107Ile, Thr137Thr, Ala143Ala
		--	8	Ile82Ile

**Table 2:** VKORC1 mutations and polymorphisms found in *Mus musculus *from different geographic areas in Germany and from Terceira, Azores.

**Geographic** **area**	**Amino acid** **substitutions**	**No. of samples**	**Silent** **mutations**
Berlin	Glu37Gly	12	--
Lower Saxony	Glu37Gly	1	--
Westphalia	Arg58Gly	13	--
	Arg12Trp, Ala26Ser, Ala48Thr, Arg58Gly, Arg61Leu	7	Glu37Glu
	Arg12Trp, Ala26Ser, Ala48Thr, Arg61Leu	2	Glu37Glu
Rhineland	Leu128Ser	17	--
	Tyr139Cys	1	--
Azores	Tyr139Cys	1	--

**Figure 1:**
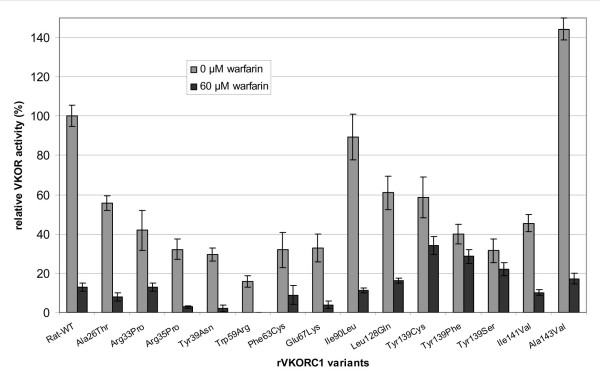
**Relative VKOR activities of different rat VKORC1 variants after recombinant expression in HEK293 cells**. Light grey bars indicate basal VKOR activities of the variants in the absence of warfarin; dark grey bars show VKOR activities after addition of 60 μM warfarin. The specific activity of the recombinant rat wild-type enzyme (WT) was 1.0 ± 0.37 nmoles/mg protein × h and was set to 100% for comparison. Standard deviations are indicated by black error bars.

**Figure 2:**
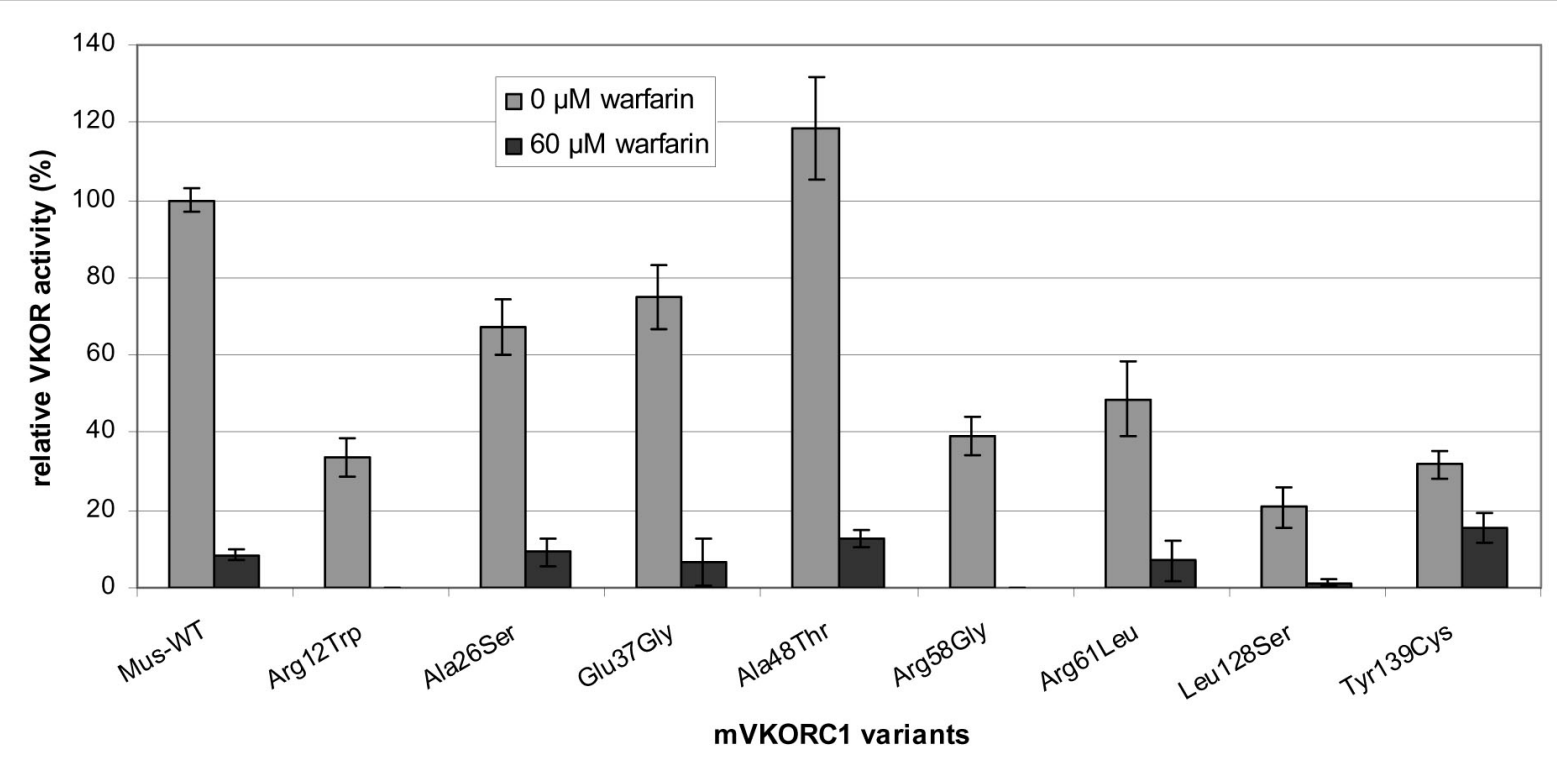
**Relative VKOR activities of different mouse VKORC1 variants after recombinant expression in HEK293 cells**. The specific activity of the recombinant murine wild-type enzyme was 0.65 ± 0.15 nmoles/mg protein × h. This value was set to 100% for comparison. For further details see legend to Figure 1.

A recombinant expression system in HEK293 cells was used to assay for the VKOR activities of the mutant proteins *in vitro *and to study their sensitivity to warfarin (Fig. 1 and 2). Transfection efficiency was checked by Western blotting using the anti-FLAG antibody M2 and was found to be similar for all VKORC1 protein variants (data not shown).

### VKORC1 sequence variants

Some of the observed nucleotide variants do not result in amino acid substitutions and are supposedly silent mutations. Several of these SNPs (e.g. Ile107Ile, Thr137Thr) were detected in rats from USA, Argentina, Thailand, Indonesia and the Azores but not observed in European, Korean or Japanese rats (Table 1). This may be explained by independent population founder effects. The Ile82Ile variant occurred at high frequency in rats from all continents except Africa. This SNP may, thus, be an ancestral variant or may have arisen several times independently.

**Figure 3:**
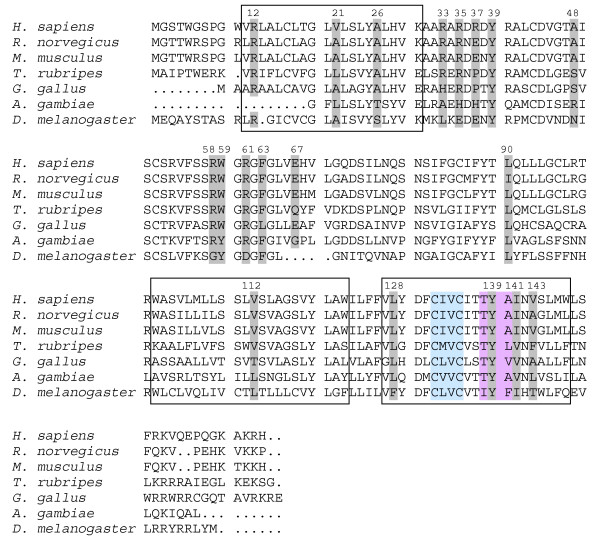
**Alignment of VKORC1 proteins of different species**. Amino acid substitutions which could be detected in the examined rats and mice are highlighted by grey shading. Black boxes indicate the predicted transmembrane domains (according to the topology model of Tie et al., 2005 [30]). Blue shaded amino acid residues refer to the CXXC motif which is supposed to be the active site of the VKORC1 protein. The TYA motif which is highlighted in pink is the proposed warfarin binding site [25].

Of the variants which do cause amino acid substitutions, isoleucine-90 was found to be substituted by leucine and alanine-143 by valine in some rats from both North and South America and/or Southeast Asia. However, in the human VKORC1 protein these latter amino acids represent the wild-type (Fig. 3). Further, they are functionally conservative amino acid substitutions and show similar or even higher VKOR activities than the wild-type *in vitro *(Fig. 1). Thus, these substitutions can be considered functionally neutral variants. Isoleucine-141 was substituted by valine in some rats from Indonesia. It reduces VKOR activity to the half of the wild-type activity *in vitro*. In the VKORC1 of other species, *e.g. T. rubripes, G. gallus *and *A. gambiae *(Fig. 3) valine represents the wild-type at position 141. The effect of the Ile141Val mutation in combination with the change from alanine-143 to valine which was observed in one rat from Indonesia remains to be investigated.

Other amino acid substitutions were shown to only have a slight effect on VKOR activity in the *in vitro *assay. The glutamate residue at position 37 was found to be substituted by glycine in 12 mice from Berlin, Germany. This glutamate is not highly conserved, not even in other vertebrates, e.g. the human VKORC1 has an arginine at this position (Fig. 3). VKOR activity assays of the Gly37 variant revealed a basal VKOR activity of 75% compared to the wild-type protein. The Gly37 variant is inhibited by warfarin in a manner comparable to the wild-type murine VKORC1 protein (Fig. 2). Thus, Gly37 rather seems to be a polymorphism in the murine VKORC1 with only a minor effect on protein function.

### VKORC1 mutations

English rats showed the greatest diversity of mutations in the VKORC1 gene. Three already described mutations could be detected in rats trapped in different English counties: the Tyr139Cys substitution was found in rats from Gloucestershire, Norfolk and Lincolnshire, the Tyr139Ser mutation in rats from Shropshire and the Leu128Gln substitution could be shown in rats from Lancashire. All three mutations confer a moderately reduced VKOR activity and are resistant to warfarin inhibition to a variable degree (Fig. 2 and [14]). In two confirmed warfarin-resistant rats from Nottinghamshire an Arg33Pro substitution was observed. VKOR activity of the Arg33Pro variant was reduced to 42% of wild-type activity. A Phe63Cys substitution was detected in rats from Cambridge, including two rats with an additional heterozygous point mutation leading to either an Ala26Thr or a Tyr39Asn amino acid exchange. While Ala26Thr has – similar to Ala26Ser – only a moderate effect on VKOR activity with a reduction to approximately 56% of wild-type activity, the Phe63Cys and the Tyr39Asn substitutions reduce the VKOR activity to about 30% of normal. Since both amino acids Phe63 and Tyr39 are highly conserved in vertebrates and also in the mosquito, a substitution of these amino acids is expected to have an influence on protein function.

VKOR activity measurements of the Glu67Lys variant (observed in six rats from Japan) showed a reduced vitamin K epoxide turnover of about 33% compared to the wild-type protein. However, none of these novel mutations seems to have an effect on warfarin sensitivity *in vitro*.

The most drastic effect on VKOR activity was observed for the Trp59Arg substitution. Only 16% residual VKOR activity could be measured after recombinant expression of this VKORC1 mutant. Therefore, warfarin resistance could not be tested for *in vitro*. The Trp59Arg mutation was found both in heterozygous and homozygous genotypes in rats from Argentinean poultry farms. Anticoagulants have been intensively used in these agricultural areas for rodent pest control. In contrast, rats trapped on a waste deposit in Buenos Aires showed no amino acid substitutions in the VKORC1 protein. Given the low *in vitro*-activity of the Trp59Arg substitution, one could expect animals homozygous for this mutation to suffer from a deficiency of vitamin K-dependent coagulation factors with an increased bleeding risk even without warfarin uptake. In theory, these rats could be rescued by a much higher vitamin K intake as has been described for some warfarin resistant laboratory strains [15-17]. Field investigations of the relative fitness of warfarin-resistant rats on a farm in Shropshire (UK) showed that when selection pressure from anticoagulant use was removed, the frequency of the warfarin resistance gene fell from 80 to 33% over 18 months [18].

Seven mice trapped in Münsterland, Germany, carried the same 5 amino acid substitutions each (Table 2), thus posing the question which conferred warfarin resistance *in vivo*, either independently or in combination. Recombinant VKOR activities of the variants Ala26Ser and Ala48Thr – when studied separately – were determined at 67% and 118% of normal, respectively. While Ala26 is conserved in most vertebrates, there is a serine at this position in the fruitfly and a threonine in the mosquito VKORC1 (Fig. 3). Similarly, Ala48 is only conserved in higher vertebrates and is substituted by serine in chicken or by arginine in the mosquito (Fig. 3).

Expression of the variants Arg12Trp, Arg58Gly and Arg61Leu revealed a strong influence on vitamin K epoxide reduction compared to the wild-type protein. All three substitutions reduced VKOR activity to 33%, 39% and 49% of the mouse wild-type, respectively. However, none of these substitutions on their own, conferred a significant warfarin resistance in the *in vitro *assay (Fig. 2). The Arg58Gly substitution was also found as the only variant – either heterozygous or homozygous – in a total of 13 mice from Germany. The same mutation was described before in a warfarin-resistant patient from Norway (Table 3) [3]. This patient required a weekly dose of 220–250 mg warfarin for an effective anticoagulation therapy (normal dose: 10–60 mg per week). Although warfarin resistance is clearly evident for this patient *in vivo *it could not be demonstrated *in vitro *for the Arg58Gly variant nor for the other mutations detected in mice, except for the Tyr139Cys mutation found in wild mice from the Rhineland, Germany and from Terceira, Azores. This mutation has previously been found in anticoagulant-resistant breeding colonies derived from wild mouse populations in England [3,14]. These findings suggests a wide distribution and some selective advantage for this resistance-mediating mutation in mice.

**Table 3:** Overview of mutations found in VKORC1 in rats, mice and humans.

***Rattus norvegicus***	**NCBI_ss#**	***Mus musculus*/** ***Mus domesticus***	***Homo*** ***sapiens***	**Reference**
Arg12Arg	107794658	--	--	this study
--	--	Arg12Trp	--	this study
Ala21Thr	107794659	--	--	this study
Ala26Thr	107794660	Ala26Ser	--	this study
--	--	--	**Val29Leu**	[3]
**Arg33Pro**	107794661	--	--	[13]; this study
**Arg35Pro**	107794662	--	--	[14]; this study
--	--	Glu37Gly	--	this study
Tyr39Asn	107794663	--	--	this study
--	--	--	**Val45Ala**	[3]
--	--	Ala48Thr	--	this study
Ser56Pro	107794664	--	--	this study
--	--	Arg58Gly	**Arg58Gly**	[3]; this study
**Trp59Arg**	107794665	--	--	this study
--	--	Arg61Leu	--	this study
--	--	--	**Val66Met**	[28]
Phe63Cys	107794666	--	--	this study
Glu67Lys	107794667	--	--	this study
Ile82Ile	107794668	--	--	this study
Ile90Leu	107794669	--	--	this study
Leu94Leu	107794670	--	--	this study
Ser103Ser	107794671	--	--	this study
Ile107Ile	107794672	--	--	this study
Val112Leu	107794673	--	--	this study
**Leu120Gln**	107794674	--	--	[14]
**Leu128Gln**	107794675	**Leu128Ser**	**Leu128Arg**	[3,14]; this study
Thr137Thr	107794676	--	--	this study
**Tyr139Phe**	107794677	--	--	[14,29]; this study
**Tyr139Cys**	107794678	**Tyr139Cys**	--	[3,14]; this study
**Tyr139Ser**	107794679	**Tyr139Ser**	--	[14]; this study
Ile141Val	107794680	--	--	this study
Ala143Ala	107794681	--	--	this study
Ala143Val	107794682	--	--	this study

Mutations in bold indicate where resistance was proven in rats or mice, or dosing problems in humans were reported. All sequence variants found in rats were submitted to dbSNP at NCBI  and are indicated by their distinct NCBI_ss# numbers.

In the *in vitro *assay used to measure VKOR activity, surprisingly most mutations were rather sensitive to inhibition by warfarin. In the assay used, transfected cells are lysed with relatively high concentrations of detergent (0.5% CHAPS) and, thus, the hydrophobic membrane environment that is required for VKOR activity [19] may have been impaired. These results are, however, consistent with earlier studies on VKOR activity in liver microsomal preparations from Scottish [20,21] and Chicago [22] warfarin-resistant strains of rats that have now been shown to be homozygous for the Leu128Gln and Arg35Pro substitutions, respectively, in VKORC1 [14]. Those studies used intact liver microsomal preparations to determine the kinetic parameters of VKOR activity and its sensitivity to warfarin inhibition. In both resistant strains, the enzyme parameters were found to be very similar to those of warfarin-susceptible laboratory rats. There was evidence, however, that warfarin was less tightly bound to the microsomal preparations from resistant strains and inhibition of VKOR was reversible [21,22]. Similar work on hepatic microsomal preparations from a resistant mouse strain – now known to carry a Leu128Ser substitution in VKORC1 [14] – showed the same activity and warfarin sensitivity of VKOR as in susceptible mice [23].

So far, *in vivo- *and *in vitro-*data on warfarin resistance match only for substitutions at amino acid position 139 of the VKORC1 protein. Tyr139Cys was identified in this study in two rats from Hungary as well as in five wild-caught English rats and was reported before in rats from Germany (Münsterland), Denmark and from England [14]. In this earlier report, rats were characterized as warfarin-resistant by the blood clotting response test (BCR). The mutation Tyr139Phe was found in six Korean rats in this study, and had been detected before in French and Belgian rats [14]. Warfarin has been extensively used and supplied free of charge to the citizens in Korea since the 1950's. The Tyr139Ser substitution which could be detected in two rats from Shropshire, England had been described in a laboratory strain of rats derived from wild animals trapped in the same area 30 years before [24]. In the recombinant expression system, basal VKOR activities of the Tyr139Cys, the Tyr139Phe and the Tyr139Ser variants, respectively, were lower than of the rat wild-type protein but in the presence of 60 μM warfarin a significantly higher residual VKOR activity was retained (Fig. 1). Therefore, rats bearing either of the Tyr139 mutations should have an enormous selective advantage in warfarin-exposed areas. We have speculated earlier that Tyr139 may be part of the warfarin binding site of VKORC1 [25].

The synopsis of mutations in VKORC1 (Table 3) shows that amino acid substitutions are observed all over the gene with foci on positions 29 to 48 in exon 1, positions 58 to 67 in exon 2, and 120 to 143 in exon 3. The substitutions Arg33Pro, Arg35Pro, Trp59Arg, Leu120Gln, Leu128Gln, Leu128Ser, Tyr139Cys, Tyr139Phe and Tyr139Ser have been associated with positive results from *in vivo *resistance testing (by blood clotting response or no-choice feeding tests) in rodents; the replacements Val29Leu, Val45Ala, Arg58Gly, Val66Met and Leu128Arg have been correlated with an increased coumarin requirement for maintaining normal clotting times in human thrombophilic patients. These changes in the VKORC1 protein could, therefore, be associated with the *in vivo *warfarin resistance phenotype. For amino acid substitutions of Tyr139, this can be followed by the *in vitro *resistance of recombinant VKORC1 activity to warfarin (Fig. 1). For the remaining substitutions, their relation to *in vivo *resistance remains to be demonstrated.

## Conclusion

In conclusion, we have identified 18 novel amino acid substitutions and five known mutations, as well as eight silent sequence variants in the *VKORC1 *gene of wild rats and mice trapped in anticoagulant-exposed areas on four continents. Although not all of these rodents were classified as warfarin-resistant by BCR or laboratory feeding tests, most of them had survived in an anticoagulant-treated habitat. In a recombinant expression system, VKOR activity was substantially reduced by most VKORC1 variants compared to the wild-type proteins but reduced sensitivity to warfarin inhibition could not be demonstrated in this assay except for the Tyr139 variants. In the Welsh-type resistant laboratory rat strain carrying the Tyr139Ser substitution, low VKOR activity (25–50% in comparison to the susceptible rats) was found to be associated with an enhanced (> 10-fold) dietary vitamin K requirement [17]. It is not known whether a similar dietary compensation would be possible in the wild. Further studies are needed to establish the mechanism by which mutations in the VKORC1 gene lead to warfarin resistance.

## Methods

### Animals

Wild rats (*Rattus norvegicus*) were trapped in known or suspected resistance areas in Hungary, England, Indonesia, Korea, Japan, Thailand, South Africa, the USA, Argentina and one island of the Azores. Rats from Chicago, Illinois came from a breeding colony at Genesis Laboratories, Colorado. The founder population of this breeding colony had been acquired in July 2000 by live-trapping rats in Chicago [26]. Three past GLP studies at Genesis Laboratories have identified about half of the animals from the same location in Chicago to be resistant to warfarin by feeding tests [27].

Wild mice (*Mus musculus*) came from four areas of Germany (Lower Saxony, Berlin, Westphalia and Rhineland) and from the island of Terceira, Azores.

Samples were collected from 2005 through 2007, except for the English rat samples from Gloucestershire, Lancashire, Lincolnshire, Norfolk, Nottinghamshire and Shropshire, which were from rats trapped in 1994 through 1996 and stored frozen.

DNA was extracted from different tissues using standard procedures. The warfarin resistance status of the rodents had been checked in a few cases using blood clotting response [13] or feeding tests [27], but was unknown in most cases.

All sampling was done under licence and authorization of the regional rodent pest control authorities in accordance with national legislation.

### ARMS-PCR and sequence analysis

After DNA extraction, all samples were tested by an allele-specific PCR (ARMS) for the presence of the Tyr139Cys mutation which is the prevalent mutation in warfarin resistant rats and mice from North-Western Europe [14].

Samples which were negative in the ARMS test or showed an abnormal band pattern (divergent from the Tyr139Cys band pattern) were sequenced for the three exons of the VKORC1 gene on an automatic sequencer CEQ8000, Beckman Coulter. Primer sequences are available on request and conditions are as described by Pelz et al., 2005 [14].

### Cloning and mutagenesis

Rat and mouse VKORC1 cDNAs were cloned into the pCEP4 vector (Invitrogen, Carlsbad, California) and a N-terminal FLAG tag (M-DYKDDDDK) was added to the construct for monitoring of the transfection efficiency by Western blot analysis. Mutations were introduced into the pCEP4-VKORC1 construct with the QuikChange mutagenesis kit (Stratagene, Amsterdam, NL) according to the manufacturer's instructions.

### Expression and VKOR activity assay

For the recombinant expression of rat and mouse VKORC1 variants HEK293-EBNA cells (Invitrogen, Karlsruhe, Germany) were used as described earlier [25]. The VKOR enzymatic activity of the various VKORC1 proteins was measured in whole-cell extracts before and after addition of different warfarin concentrations as described by Rost et al., 2005 [25]. Aliquots of whole-cell protein extracts were separated on 4–12% NuPAGE gels (Invitrogen, Karlsruhe, Germany) according to the manufacturer's instructions and blotted onto nitrocellulose membranes (Whatman Schleicher & Schuell, Dassel, Germany). Monoclonal FLAG M2 antibody (Sigma-Aldrich) was used for immuno-staining of the Western blots.

## Authors' contributions

SR performed the mutation screening and cloning, coordinated the VKOR activity studies and drafted the manuscript. HJP conceived of the study, coordinated the collection of all rat and mice samples and the performance of the ARMS test and helped to draft the manuscript. SM carried out the mutagenesis and VKOR activity assays. ADM carried out the resistance-testing of the UK-rats and ADM, VL, KJS and TJ collected numerous rat and mouse samples in their countries and participated in the design of the study. JO and CRM contributed substantially to the conception and design of the study, to the interpretation of data and revised the manuscript. All authors read and approved the final manuscript.
